# Artificial intelligence across the cancer care continuum

**DOI:** 10.1002/cncr.70050

**Published:** 2025-08-14

**Authors:** Irbaz Bin Riaz, Muhammad Ali Khan, Travis J. Osterman

**Affiliations:** ^1^ Division of Hematology/Oncology Department of Medicine Mayo Clinic Phoenix Arizona USA; ^2^ Department of Artificial Intelligence and Informatics Mayo Clinic Rochester Minnesota USA; ^3^ Department of Biomedical Informatics Vanderbilt University Medical Center Nashville Tennessee USA; ^4^ Department Medicine Vanderbilt University Medical Center Nashville Tennessee USA

**Keywords:** artificial intelligence (AI), cancer screening, cancer survivorship, end‐of‐life care, medical oncology, radiation oncology, surgical oncology

## Abstract

Artificial intelligence (AI) holds significant potential to enhance various aspects of oncology, spanning the cancer care continuum. This review provides an overview of current and emerging AI applications, from risk assessment and early detection to treatment and supportive care. AI‐driven tools are being developed to integrate diverse data sources, including multi‐omics and electronic health records, to improve cancer risk stratification and personalize prevention strategies. In screening and diagnosis, AI algorithms show promise in augmenting the accuracy and efficiency of medical image analysis and histopathology interpretation. AI also offers opportunities to refine treatment planning, optimize radiation therapy, and personalize systemic therapy selection. Furthermore, AI is explored for its potential to improve survivorship care by tailoring interventions and to enhance end‐of‐life care through improved symptom management and prognostic modeling. Beyond care delivery, AI augments clinical workflows, streamlines the dissemination of up‐to‐date evidence, and captures critical patient‐reported outcomes for clinical decision support and outcomes assessment. However, the successful integration of AI into clinical practice requires addressing key challenges, including rigorous validation of algorithms, ensuring data privacy and security, and mitigating potential biases. Effective implementation necessitates interdisciplinary collaboration and comprehensive education for health care professionals. The synergistic interaction between AI and clinical expertise is crucial for realizing the potential of AI to contribute to personalized and effective cancer care. This review highlights the current state of AI in oncology and underscores the importance of responsible development and implementation.

## Abstract 2

This review highlights the expanding role of artificial intelligence across the cancer care continuum, from risk prediction to end‐of‐life care. It emphasizes both the promise of artificial intelligence to personalize oncology and the critical need for responsible, interdisciplinary integration into clinical practice.

## INTRODUCTION

Artificial intelligence (AI) is poised to be a transformative technology that promises to change the way we care for patients with cancer. Our evolving understanding of cancer biology, coupled with the exponential increase in the availability of genomic information, electronic health records (EHRs), and medical imaging, has created a unique and timely opportunity to leverage the power of AI to revolutionize cancer care. The potential of AI to improve cancer care spans the entire spectrum of the disease, from prevention and early detection to treatment, survivorship, and end‐of‐life care.^1^, ^2^

AI‐powered tools are being developed and implemented to enhance our ability to assess individual cancer risk, improve the accuracy and efficiency of screening programs, refine the precision of diagnostic procedures, personalize treatment strategies based on individual patient characteristics, and provide comprehensive support to patients and their families throughout their cancer journey. For example, AI algorithms can analyze genomic data to identify individuals at high risk for developing specific cancers, enabling targeted prevention strategies. AI‐powered image analysis tools can assist radiologists and pathologists in detecting subtle abnormalities in medical images, improving the accuracy and speed of cancer diagnosis. AI can also be used to develop personalized treatment plans based on a patient's unique genetic profile and clinical history, maximizing the chances of treatment success and minimizing the risk of side effects.

This review article aims to provide an overview of AI's current and potential applications in oncology practice, encompassing the entire cancer care continuum. In addition to exploring the diverse applications of AI in oncology, this review also addresses the key challenges and ethical considerations associated with implementing AI in cancer care. These include the critical need for rigorous AI algorithm validation to ensure accuracy, reliability, and clinical utility. We also discuss the importance of addressing ethical concerns, such as data privacy and security, potential algorithm bias, and the need for transparency and explainability in AI‐driven decision making. Furthermore, we emphasize the crucial role of seamless integration of AI tools into existing clinical workflows to facilitate their effective adoption by health care professionals. Finally, we highlight the importance of interdisciplinary collaboration between clinicians, researchers, data scientists, technology developers, and policymakers to overcome these challenges and fully realize the transformative potential of AI in oncology. Our goal is to provide practicing physicians, researchers, and leaders in oncology and related fields with a comprehensive and nuanced understanding of the current state of the art of AI in oncology and the future directions and immense potential of this rapidly evolving field. By fostering informed discussion and promoting collaborative efforts, we hope to accelerate the responsible and effective implementation of AI in cancer care, ultimately improving outcomes and quality of life for all patients with cancer and their families.

## PREVENTION AND EARLY INTERCEPTION

Cancer prevention represents a critical frontier in the fight against this disease. Identifying high‐risk individuals and improving early detection can significantly affect patient outcomes by personalizing screening. AI is a promising tool in this domain and has the potential to revolutionize how we approach cancer prevention. AI's ability to analyze vast data sets, identify complex patterns, and learn from experience is paving the way for more personalized and effective prevention strategies (Table 1).^3^, ^15^, ^20^

**TABLE 1:** Artificial intelligence in cancer prevention and diagnosis.^a^

Modality	Study	Cancer type	Task	Performance metric	Performance
Prevention and early detection	Kakileti 2020^3^	Breast	Pre‐screening prediction of cancer risk (Thermalytix risk score)	AUC	AUC: 0.89
	Arefan 2020^4^	Breast	Prediction of breast cancer risk from normal mammograms	AUC	AUC: 0.73
	Badre 2021^5^	Breast	Estimation of polygenic risk score for patients	AUC	AUC (DNN): 0.67
	Yala 2021^6^	Breast	Prediction of breast cancer risk from mammograms	C‐index, AUC	C‐index (highest): 0.81 (95% CI, 0.79–0.82) AUC (highest): 0.90 (95% CI, 0.89–0.92)
	Ha 2019^7^	Breast	Pixel‐wise prediction of breast cancer risk from mammograms	Accuracy	Accuracy: 0.72 (95% CI, 69.8–74.4)
	Hernstrom 2025^8^	Breast	A randomized trial to assess cancer detection on mammography and reduction in screen‐reading workload	Proportion ratio for cancer detection, reduction in screen‐reading workload	Proportion ratio (invasive cancer): 1.24 (95% CI, 1.05–1.51) Proportion ratio (in situ cancer): 1.51 (95% CI, 1.03–2.19) Screen‐reading workload reduction: 44.2%
	Levi 2024^9^	Lung	Prediction of lung cancer risk from electronic medical records	Accuracy, sensitivity, PPV	Accuracy: 71.2% Sensitivity: 69% PPV: 74%
	Yeh 2021^10^	Lung	Prediction of lung cancer risk from electronic medical records	AUC, PPV	AUC: 0.90 PPV: 14.3%
	Mikhael 2023^11^	Lung	Prediction of lung cancer risk from low‐dose chest computed tomography	AUC	AUC (highest): 0.94 (95% CI, 0.91–1.00)
	Wang2024^12^	Cervical	AI cancer screening system for grading cervical cytology	AUC, sensitivity, specificity, accuracy	AUC: 0.947 Sensitivity: 0.946 Specificity: 0.890 Accuracy: 0.892
	Xu 2023^13^	Colorectal	A randomized trial comparing AI‐assisted colonoscopy with conventional colonoscopy for adenoma detection in an asymptomatic population	ADR	ADR (AI vs. conventional): 39.9% vs. 32.4% (*p* < .001)
	Placido 2023^14^	Pancreatic	Prediction of pancreatic cancer from disease trajectories	AUC	AUC (best): 0.88
	Nazha 2021^15^	Myelodysplastic syndromes	Personalized prediction of survival and leukemic transformation	C‐index	C‐index (survival): 0.71 (95% CI, 0.73–0.75) C‐index (leukemic progression): 0.84 (95% CI, 0.73–0.75)
Diagnosis	Harder 2024^16^	Prostate	Tumor and Gleason grade detection from histopathology images	Sensitivity (tumor detection), specificity detection (tumor detection), quadratic kappa range (Gleason grade detection)	Sensitivity: 0.97 Specificity: 0.97 Quadratic kappa range: 0.77–0.78
	Too 2024^17^	Lung	Detection of lesions and predicting biopsy needle trajectories from CT scans	AUC (lesion detection), feasibility (needle path), actual path matching	AUC: 97.4% (95% CI, 96.3%–98.2%) Feasibility: 85.3% Actual path matching: 82%
	Darbandsari 2024^18^	Endometrial	Prediction of molecular cancer subtype from histopathology images	Mean balanced accuracy, AUC	Mean balanced accuracy: p53abn, 89.4%; NSMP, 79.8% AUC: p53abn, 0.95; NSMN, 0.88
	Abbas 2019^19^	Gastric; colorectal	Cancer grading from histopathology images	Accuracy, macro F1 score, quadratic weighted kappa	Accuracy (highest for colorectal cancer): 88.55 ± 0.71 Accuracy (gastric cancer): 87.32 ± 0.384 F1 (highest for colorectal cancer): 0.865 ± 0.710 F1 (gastric cancer): 0.834 ± 0.007 Kappa (highest for colorectal cancer): 0.952 ± 0.007 Kappa (gastric cancer): 0.936 ± 0.010

Abbreviations: ADR, adenoma detection rate; AI, artificial intelligence; AUC, area under the curve; CI, confidence interval; CT, computed tomography; DNN, deep neural network; NSMP, no specific molecular profile; p53abn, p53 abnormality; PPV, positive predictive value.

^a^ The application of AI algorithms in cancer screening, detection, and diagnosis.

One of the most promising applications of AI in cancer prevention lies in its ability to assess and predict individual cancer risk. Algorithms can integrate diverse data sources, including multi‐omic information, EHRs, lifestyle factors (e.g., smoking, diet, physical activity), environmental exposures, and family history, to generate personalized risk profiles.^3^, ^15^, ^20^ For example, AI‐powered tools can analyze mammography images in conjunction with genetic markers and clinical data to predict a woman's risk of developing breast cancer.^4^, ^5^ Similarly, AI systems can assess an individual's risk for lung cancer based on their smoking history, exposure to carcinogens, and other relevant factors.^9^, ^10^ A recent study demonstrated the power of AI in predicting pancreatic cancer risk by analyzing disease trajectories from EHRs. Their deep learning models, particularly using Transformer architectures, achieved remarkable predictive performance (area under the receiver operating characteristic curve = 0.88) for identifying high‐risk patients up to 36 months before diagnosis by analyzing temporal sequences of disease codes from 6 million Danish patients and 3 million US veterans. This work represents a significant advance in using AI for early detection of aggressive cancers that typically present late.^14^ These predictive models can empower health care providers to identify high‐risk individuals who may benefit from more intensive screening, chemoprevention, or lifestyle modifications. However, it is crucial to acknowledge the ethical considerations surrounding AI‐driven risk prediction. Potential biases in the data used to train these algorithms must be carefully addressed to ensure equitable access to preventive interventions.^21^ Furthermore, responsible data handling and privacy protection are paramount.^22^ Pathologists, with their expertise in interpreting tissue samples and understanding disease mechanisms, play a crucial role in developing and validating AI‐driven risk‐prediction models that incorporate pathologic data. Pathologists and radiologists, with their expertise in interpreting tissue samples and medical images, respectively, play a crucial role in developing and validating AI‐driven risk‐prediction models. Radiologists ensure imaging data quality and consistency, reducing biases from heterogeneous data sets, whereas pathologists refine AI algorithms through accurate annotations and histopathologic validation. Their collaboration with AI developers is essential for refining these tools, ensuring clinical applicability, and maintaining high diagnostic accuracy and patient care standards.

## SCREENING AND EARLY DIAGNOSIS

An accurate and timely cancer diagnosis is essential for effective treatment planning and improved patient outcomes. Radiology plays a pivotal role by providing noninvasive imaging of tumors, and AI is augmenting the expertise of radiologists by streamlining image interpretation and improving diagnostic accuracy (Table 1). AI algorithms analyze various imaging modalities, including computed tomography (CT) scans, magnetic resonance images (MRIs), and positron‐emission tomography scans, to detect subtle abnormalities that may indicate cancer, improving sensitivity and reducing false‐negative rates.

Expanding on AI's transformative role in radiology, one particularly effective application is that for mammography, in which AI enhances breast cancer detection through advanced image analysis. AI‐powered algorithms assist in analyzing mammograms, identifying subtle patterns and features indicative of early stage breast cancer. A landmark trial involving 105,934 participants demonstrated that AI‐assisted screening achieved a 29% higher cancer detection rate (6.4 vs. 5.0 per 1000 screened), primarily identifying early stage cancers and reducing radiologist workload by 44% without increasing the false‐positive rate compared with standard double reading.^8^

AI is also advancing lung cancer screening by improving the detection of small lung nodules on CT scans. Identification of these nodules is essential for guiding biopsy decisions and enabling early diagnosis, which significantly improves the chances of successful curative treatment. In addition, AI is enhancing the accuracy and efficiency of tissue acquisition. AI‐powered image‐guidance systems assist interventional radiologists in precisely targeting lesions during biopsies, reducing sampling errors and improving diagnostic yield. For instance, AI algorithms can analyze real‐time imaging data from CT scans or MRIs to enhance needle placement accuracy for lung and prostate biopsies, thereby increasing the detection rate of clinically significant cancers and reducing the need for repeat procedures.^16^, ^17^

Once tissue samples are obtained, pathologists play a central role in diagnosing cancer through microscopic examination. AI is significantly enhancing pathology by automating histopathology image analysis, assisting pathologists in identifying cancer subtypes, grading tumors, and assessing disease progression.^18^, ^19^, ^23^, ^24^ AI‐powered tools can analyze digital pathology slides, detecting subtle cellular features and biomarkers that may otherwise be overlooked, thus improving diagnostic precision (Table 1). For example, AI is being applied in cytopathology to analyze Papanicolaou smears for the early detection of cervical cancer, help identify prostate cancer, and provide Gleason scores. Several US Food and Drug Administration‐approved, AI‐powered digital pathology solutions are now available, such as Paige Prostate,^25^ which identifies areas in prostate biopsy images likely to harbor cancer, and Ibex Galen Prostate,^26^ which assists in prostate cancer detection and grading.

Beyond imaging‐based and tissue‐based diagnostics, AI is playing an increasingly important role in molecular diagnostics by complementing these traditional methods with genomic and molecular insights, enabling a more comprehensive approach to cancer detection and treatment planning. AI from histopathology slides can assess tumor microenvironment and identify genetic mutations driving cancer growth and predict responses to targeted therapies or immunotherapy.^27^, ^28^ Deep‐learning approaches have been used to analyze tumor heterogeneity, predicting tumor evolution and resistance mechanisms and identifying prognostic biomarkers. Examples include analyses of histopathology slides to classify molecular subtypes of lung and colorectal cancer, evaluation of tumor microenvironments in renal cell carcinoma by quantifying immune cell infiltration, aiding in immunotherapy response predictions, detecting genetic alterations, such as EGFR and KRAS mutations in lung cancer and BRCA mutations in ovarian cancer, all of which hold significant predictive value and influence treatment decisions.

Although AI is improving cancer screening and early diagnosis, its success depends on collaboration between AI systems and medical professionals.^12^, ^13^ AI enhances efficiency by reducing workload and expediting image analysis, yet expert oversight remains essential to ensure accuracy, interpret complex cases, and provide clinical context. Radiologists and pathologists play a crucial role in integrating AI findings into practice, ensuring that these technologies enhance—not replace—expert clinical judgment. AI tools enable radiologists to work more efficiently, focusing on complex cases and improving overall patient care while reducing turnaround time for diagnostic interpretations.

## TREATMENT

Effective cancer treatment requires a multifaceted approach, often involving combinations of surgical intervention, radiation therapy, and systemic therapies. AI is being integrated into each of these treatment modalities, offering the potential for more precise, personalized, and effective cancer care (Table 2).^3^, ^15^, ^20^

**TABLE 2:** Artificial intelligence in cancer treatment.^a^

Treatment type	Study	Cancer type	Task	Performance metric	Performance
Surgical treatment	Auffenberg 2019^29^	Prostate	Prediction of prostatectomy outcomes from the outcomes of patients with similar clinical features	AUC	AUC, 0.81
	Park 2020^30^	Colorectal	Prediction of anastomotic complication in laparoscopic surgery from ICG angiography	F1 score increase	F1 increase (T_1/2max_): 31% F1 increase (TR): 8% F1 increase (RS): 8%
	Sharma 2025^31^	Bladder	Correlation of preoperative CT images with 90‐day postradical cystectomy complications	OR (major complications)	OR (higher vs. lower skeletal mass index): 0.75 (*p* = .008) OR (higher vs. lower adipose tissue): 1.93 (*p* = .008)
	Lyuksemburg 2023^32^	Multiple	Comparison of VRM and 2D images for preoperative planning in complex surgical oncology cases	Concordance with operative plan	Concordance (operating surgeon): 92% vs. 54% Concordance (consulting surgeon): 69% vs. 23%
Radiation treatment	Bohoudi 2017^33^	Pancreatic	Implement robust and fast MR‐guided adaptive radiation therapy	No. of optimizations; PTV coverage, doses to OAR	Optimizations (SMART_3CM_ vs. FULLOAR): 4 vs. 18 PTV (SMART_3CM_ vs FULLOAR): 89.4% vs. 89.4% Significant lower doses to all OARs with SMART_3CM_
	Rajendran 2025^34^	Prostate, oropharyngeal	Multimodal delineation of target volume for radiation therapy using large‐language models	DSC, IOU, HD_95_	DSC: 0.81 ± 0.10 IOU: 0.73 ± 0.12 HD_95_: 9.86 ± 9.77mm
Systemic treatment	Lippenszky 2024^35^	Melanoma, lung, genitourinary	Prediction of hepatitis, colitis, pneumonitis and 1‐year OS from her	AUC	AUC (pneumonitis): 0.739 AUC (hepatitis): 0.729 AUC (colitis): 0.755 AUC (1‐year OS): 0.752
	Jeon 2014^36^	Breast, pancreatic, ovarian	Identification of novel potential anticancer drug targets	BAC, AUC	BAC (breast): 91.05 AUC (breast): 78.46 BAC (pancreatic): 76.17 AUC (pancreatic): 77.47 BAC (ovarian): 78.38 AUC (ovarian): 79.31

Abbreviations: 2D, two‐dimensional; AUC, area under the curve; BAC, balanced accuracy; CT, computed tomography; DSC, Dice similarity coefficient; EHR, electronic health record; FULLOAR, full‐scale organ‐at‐risk (re‐)contouring; HD_95_, 95th percentile Hausdroff distance; ICG, indocyanine green; IOU, intersection over union; MR, magnetic resonance; OAR, organ at risk; OR, odds ratio; OS, overall survival; PTV, planned target volume; RS, rising slope; SMART_3CM_, stereotactic magnetic resonance‐guided adaptive radiation therapy (limited recontouring strategy for plan adaptation); T_1/2max_, time to 50% perfusion; TR, time ratio; VRM, visual reality monitoring.

^a^ The application of artificial intelligence algorithms in radiation, surgical and systemic treatment.

### Surgical treatment

Surgical intervention remains a cornerstone of cancer treatment for many solid tumors. AI is enhancing preoperative surgical planning, intraoperative navigation, and postoperative risk assessment, demonstrating an improvement in patient outcomes through several key applications.

AI‐assisted surgical planning allows surgeons to create detailed, patient‐specific surgical plans based on preoperative imaging data.^37^ AI algorithms can analyze CT scans, MRI, and other imaging modalities to generate three‐dimensional models of the tumor and surrounding anatomy, enabling surgeons to visualize the surgical field and plan the optimal surgical approach.^32^, ^38^ This is particularly valuable in complex surgical cases, such as those involving tumors near critical structures. For example, in urologic oncology, AI can assist in planning complex robotic prostatectomies, helping surgeons to preserve nerve function and improve postoperative outcomes.^29^

AI‐powered, intraoperative navigation systems provide real‐time guidance during surgery, helping surgeons to precisely locate tumors and avoid damaging healthy tissue.^37^ These systems can overlay preoperative imaging data onto the surgical field, providing surgeons with a clear view of the tumor's location and margins. In robotic surgery, AI is being used to enhance surgical dexterity and precision. AI algorithms can analyze real‐time video feeds from the surgical robot to provide surgeons with enhanced visual feedback and assist with complex surgical maneuvers. Intraoperative data, such as tissue perfusion and blood flow, can also be analyzed to provide surgeons with real‐time information about the surgical field and help them make informed decisions during the procedure.^30^ Furthermore, AI is being used to develop systems that can automate certain aspects of surgery, such as suturing or tissue dissection. Although fully autonomous robotic surgery is still in its early stages of development, AI‐assisted robotic systems are already improving surgical efficiency and precision.^39^

AI also plays a role in postoperative risk assessment. AI algorithms can analyze patient data, including preoperative clinical information, intraoperative data, and pathologic findings, to predict the risk of postoperative complications. This information can help surgeons to identify high‐risk patients and implement preventive measures to reduce the likelihood of complications. In urologic oncology, AI can be used to predict the risk of complications after radical cystectomy, helping surgeons to tailor postoperative care and improve patient outcomes.^31^ Pathologists, with their expertise in tissue analysis and understanding of tumor biology, are essential in providing the data that inform AI‐driven surgical planning and risk assessment. Their input ensures that the AI algorithms are trained on accurate and representative data, leading to more reliable and clinically relevant results.

### Radiation treatment

Radiation therapy plays a crucial role in treating many types of cancer. AI is enhancing the precision and effectiveness of radiation therapy through several key applications. AI‐powered target volume delineation automates the process of identifying the tumor and surrounding healthy tissues on CT scans and other imaging modalities. This process, which is traditionally done manually by radiation oncologists, can be time‐consuming and prone to variability. In the future, AI algorithms may be able to quickly and accurately delineate target volumes, reducing the workload on radiation oncologists and improving the consistency of treatment planning.^34^ Radiation treatment plans are also being optimized by using AI. AI algorithms can analyze patient‐specific data, including tumor location, size, and surrounding anatomy, to generate treatment plans that maximize the dose to the tumor while minimizing the dose to healthy tissues. This can reduce the risk of side effects and improve the therapeutic ratio of radiation therapy. Adaptive radiotherapy, which allows treatment plans to be adjusted during the course of radiation therapy based on changes in the tumor or surrounding tissues, is also being studied.^33^ AI algorithms can analyze daily imaging data to track tumor shrinkage and changes in patient anatomy, allowing radiation oncologists to modify the treatment plan in real time to ensure that the tumor receives the optimal dose of radiation while sparing healthy tissues. Furthermore, AI is being used to personalize radiation dosing. AI algorithms can integrate patient‐specific data, including tumor characteristics, genetic information, and clinical factors, to determine the optimal radiation dose for each individual patient. This personalized approach to radiation dosing has the potential to improve treatment outcomes and reduce the risk of side effects.

### Systemic treatment

Systemic treatments, such as chemotherapy, targeted therapy, and immunotherapy, play a vital role in treating many cancers. AI is transforming systemic treatment by enabling more personalized treatment selection, accelerating drug discovery, and improving toxicity monitoring.^35^ AI algorithms can analyze vast amounts of genomic data, clinical information, and pathologic findings to predict a patient's response to different systemic therapies.^40^ This allows medical oncologists to select the most effective treatment for each individual patient, maximizing the chances of success and minimizing the risk of side effects. AI is also accelerating the process of drug discovery and development. AI algorithms can analyze vast data sets of molecular information to identify potential drug targets and predict the efficacy of new drugs.^36^, ^41^ This can significantly reduce the time and cost associated with bringing new cancer drugs to market. AI is also playing an important role in monitoring treatment toxicity. AI algorithms can analyze patient data, including EHRs, laboratory results, and imaging data, to identify early signs of treatment‐related toxicity.^35^, ^42^ This allows medical oncologists to intervene early and adjust the treatment plan to minimize the risk of serious side effects. AI‐powered systems can also provide personalized recommendations for managing treatment‐related side effects, improving patient comfort and quality of life.^43^

## SURVIVORSHIP

As therapies improve, cancer survivorship is an increasingly important phase of cancer care because the number of individuals living beyond a cancer diagnosis continues to grow. AI has the potential to significantly improve the quality of life for cancer survivors by enhancing monitoring and surveillance, personalizing care plans, and addressing the long‐term physical and psychosocial effects of cancer and its treatment (Table 3).^3^, ^15^, ^20^

**TABLE 3:** Artificial intelligence in cancer survivorship and end‐of‐life care.^a^

Management type	Study	Cancer type	Task	Performance metric	Performance
Survivorship	Lee 2021^44^	Pancreatic	Prediction of cancer recurrence after surgery	C‐index	C‐index: 0.7738
	Ito 2024^45^	Lung	Determining the prognostic value of physical activity with wearable devices by testing association with ECOG status and 6‐month survival	AUC	AUC (ECOG): 0.818 (95% CI, 0.703–0.934) AUC (survival): 0.806 (95% CI, 0.694–0.918)
	Nunez 2024^46^	Multiple	Prediction of psychiatric consultation or counseling in patients from initial oncology consultation notes	Accuracy, AUC	Accuracy (consultation): 73.1% AUC (consultation): 0.824 Accuracy (counseling): 71.0% AUC (counseling): 0.784
End‐of‐life care	Bychkov 2018^47^	Colorectal	Prediction of outcomes from tissue samples	AUC	AUC: 0.69
	Manz 2020^48^	Gynecologic	Prediction of 180‐day mortality for outpatients from EHR	AUC	AUC: 0.89 (95% CI, 0.88–0.90)
	Manz 2024^49^	Multiple	Enhancing SIC with ML‐identified high‐risk patients	Percentage difference of patient encounters for SIC	Percentage difference: 10.1% (95% CI, 6.9%–13.8%; *p* < .001)
	Gajra 2022^50^	Multiple	Prediction of 30‐day mortality in patients	AUC	AUC: 0.86
	Gajra 2022^51^	Multiple	Enhancing PC referral based on 30‐day mortality risk prediction	Increase in PC and hospice referrals	PC increase: 15 per 1000 patients per month (91% increase) Hospice referral increase: 2.2 per 1000 patients per month (1100% increase)

Abbreviations: AUC, area under the curve; CI, confidence interval; ECOG, Eastern Cooperative Oncology Group; EHR, electronic health record; ML, machine learning; PC, palliative care; SIC, serious illness conversations.

^a^ The application of artificial intelligence algorithms in cancer survivorship and end‐of‐life care.

AI can play a crucial role in monitoring cancer survivors for recurrence or complications. AI algorithms can analyze data from EHRs, including laboratory results, imaging reports, and clinical notes, to identify patterns and trends that may be indicative of cancer recurrence or treatment‐related complications.^44^, ^52^ For example, AI systems can track changes in tumor markers or analyze imaging data to detect early signs of recurrence, allowing for timely intervention.

Wearable sensor data, such as activity trackers or smartwatches, can also be integrated with AI systems to monitor patients' physical activity levels, sleep patterns, and other physiologic parameters, providing valuable insights into their overall health and well‐being.^45^ These data can help health care providers to identify individuals who may be at risk for developing long‐term side effects, such as fatigue or cardiovascular problems, and to implement preventive measures.

Survivorship care plans can also be personalized using AI. AI algorithms can analyze individual patient data, including their cancer type, treatment history, genetic information, and lifestyle factors, to generate personalized recommendations for managing long‐term side effects, improving physical function, and addressing psychosocial needs.^53^ For instance, AI can help tailor exercise programs to individual patients' abilities and preferences or provide personalized recommendations for managing pain or fatigue. AI‐powered systems can also connect cancer survivors with relevant resources and support groups, helping them to navigate the challenges of survivorship.^54^

Furthermore, AI can play a crucial role in addressing the psychosocial needs of cancer survivors. AI‐powered chatbots or virtual assistants can provide emotional support, answer questions about survivorship issues, and connect patients with mental health professionals when needed. AI algorithms can also analyze patient data to identify individuals who may be at risk for developing anxiety, depression, or other mental health conditions, allowing for early intervention and support.^46^

The expertise of medical oncologists, radiation oncologists, surgeons, pathologists, radiologists, and other specialists is crucial in developing and implementing AI‐driven survivorship care programs.^55^ Their input ensures that the AI algorithms are trained on accurate and comprehensive data with the possibility that the recommendations generated by these systems are clinically relevant and tailored to the individual needs of each cancer survivor.^56^ By leveraging the power of AI, we can improve the long‐term health and well‐being of cancer survivors, helping them to live full and productive lives after their cancer diagnosis.

## END‐OF‐LIFE CARE

End‐of‐life care focuses on providing comfort, dignity, and support to patients with advanced cancer and their families. However, because of prognostic uncertainty and optimism bias, end‐of‐life planning does not occur in a timely manner, offering suboptimal benefit to patients. AI has the opportunity to become a valuable tool to enhance end‐of‐life care by improving symptom management, predicting prognosis, supporting decision making, and providing timely support to caregivers and families (Table 3). This is the most under‐developed area in applying AI to the cancer care continuum and thus offers a great deal of opportunity.

AI can play a crucial role in optimizing symptom management for patients receiving palliative care. AI algorithms can analyze patient data, including vital signs, medication records, and symptom reports, to identify patterns and trends that may indicate changes in a patient's condition.^50^, ^51^ This allows health care providers to proactively address emerging symptoms, such as pain, nausea, or shortness of breath, and to adjust treatment plans accordingly. AI‐powered systems can also provide personalized recommendations for managing symptoms, helping patients to achieve the best possible quality of life during this challenging time.

The prognosis for patients with advanced cancer is also being predicted using AI. AI algorithms can analyze patient data, including clinical information, laboratory results, and imaging data, to generate more accurate and personalized predictions of disease progression and survival.^47^, ^48^ This information can help patients and their families make informed decisions about their care, including whether to pursue further treatment or focus on comfort measures. AI‐powered prognostic tools can also assist health care providers in planning end‐of‐life care, ensuring that patients receive the appropriate level of support and resources.^49^

In the future, AI could also support decision making for patients and families facing difficult choices about end‐of‐life care. AI‐powered decision‐support tools can provide patients and families with information about different treatment options, potential outcomes, and quality‐of‐life considerations. These tools could help patients and families to understand complex medical information and make informed decisions that are aligned with their values and preferences. AI can also facilitate communication between patients, families, and health care providers, ensuring that everyone is on the same page and that the patient's wishes are respected.

Furthermore, AI can provide valuable support to caregivers and families of patients receiving end‐of‐life care. AI‐powered chatbots or virtual assistants can offer emotional support, answer questions about end‐of‐life care, and connect caregivers with resources and support groups. AI algorithms could also analyze patient data to identify caregivers who may be at risk for burnout or compassion fatigue, allowing for early intervention and support. By providing caregivers with the tools and resources they need, AI can help them to provide the best possible care for their loved ones during this difficult time.

The expertise of medical oncologists, palliative care specialists, nurses, social workers, and other health care professionals is essential in developing and implementing AI‐driven end‐of‐life care programs. Their input ensures that the AI algorithms are trained on accurate and comprehensive data and that the recommendations generated by these systems are clinically relevant and tailored to the individual needs of each patient and their family. By leveraging the power of AI, we can enhance the quality of end‐of‐life care, providing patients and their families with comfort, dignity, and support during this challenging time.

## CARE DELIVERY

Cancer care today resembles the management of other chronic conditions: every patient experiences a long trial of consultations, test results, treatments, and psychosocial encounters. The administrative load of documenting, synthesizing, and acting on that information now rivals the clinical work itself. Recent advances in natural language processing (NLP), speech‐to‐text, and large language models (LLMs) have turned summarization into one of the most mature, immediately deployable AI capabilities with the potential of directly improving patient experience and system capacity.

Current LLMs can summarize, assimilate, and rephrase information from complex clinical narratives, extract salient details, and draft encounter notes that require minimal clinician editing.^57^, ^58^ When paired with retrieval‐augmented generation, open‐source LLMs have used longitudinal patient trajectories from the MIMIC‐III Clinical Database (Medical Information Mart for Intensive Care, version 3; Beth Israel Deaconess Medical Center) for clinical text summarization while preserving temporal order and causal relationships.^59^ These same models also excel at evidence distillation, positioning them as powerful tools for clinical‐decision support (CDS).^60^ Pilot studies in oncology have illustrated LLMs as CDS tools in gynecologic,^61^, ^62^, ^63^, ^64^, ^65^, ^66^, ^67^, ^68^ prostate,^58^, ^61^, ^66^, ^69^, ^70^, ^71^, ^72^, ^73^ oropharyngeal,^74^, ^75^, ^76^, ^77^, ^78^, ^79^ lung^57^, ^61^, ^66^, ^67^, ^80^ and various other cancers.^81^, ^82^, ^83^, ^84^

State‐of‐the‐art LLMs can now accept multimodal input, including live speech, and generate structured documentation in real time. This advance underpins *digital scribes* that capture the entire encounter and draft notes autonomously. Controlled and observational studies indicate that ambient documentation both shortens charting time and lowers cognitive burden. Microsoft's Nuance DAX Copilot, an Epic‐embedded tool, has reduced *time‐in‐notes* by 1 minute per visit, halved scores on the National Aeronautics and Space Administration Task Load Index, and signaled a reduction in clinician burnout.^85^ Vendor surveys suggest that 70% of clinicians report better work–life balance and clinics regain 13–26 extra appointment slots per provider per month when documentation *writes itself*.^86^ By using ABRIDGE, another ambient clinical documentation tool, 81% of clinicians reported easier documentation compared with the current workflows according to vendor surveys. A multispecialty trial at the University of Kansas Medical Center indicated higher professional satisfaction and lower cognitive load for nearly 100 clinicians already using legacy speech‐recognition tools.^87^ Leading cancer institutes are now deploying ABRIDGE across hematology and medical oncology services^88^ after pilots confirmed accurate handling of complex terminology and multilingual conversations.^89^ Beyond easing clinician workload, ambient systems automatically produce lay‐language after‐visit summaries, reinforcing education and shared decision making for patients navigating intricate oncology care plans.

Beyond point‐of‐care support, AI is increasingly automating downstream administrative workflows, such as appointment scheduling, revenue‐cycle management, and population‐health outreach. Machine‐learning scheduling platforms, such as iQueue for Infusion Centers (LeanTaaS) *level‐load* daily infusion demand, shortening patient wait times and increasing chair use.^90^ For billing, a Peterson Health Technology Institute task force report notes that an ambient scribe tech produces structured notes for preparing authorizations, submitting claims, and billing functions.^91^ An IMO Health brief further demonstrates that ambient AI can auto‐map diagnoses to standardized codes, improving billing accuracy and overall return on investment,^92^ and a separate case study of an AI‐driven charge‐capture platform documented a 15% increase in captured revenue and a 20% reduction in denials.^93^ Finally, by writing discrete EHR fields, such as diagnosis, regimen, and next test date, AI systems enable population‐health dashboards to flag overdue survivorship or surveillance visits and trigger timely patient outreach.^94^, ^95^

## EVIDENCE GENERATION AND SYNTHESIS

Contemporary clinical practice guidelines are informed by high‐quality, up‐to‐date evidence generated through clinical trials and synthesized through systematic reviews and meta‐analyses. Evidence generation requires protocol drafting, regulatory submission, eligibility screening, enrolment, and longitudinal outcome capture. Evidence synthesis includes literature search, study selection, data extraction, quality appraisal, and statistical pooling. When performed manually, these processes become time‐consuming, resource‐intensive, and prone to human error and variability, rendering the evidence outdated by the time a guideline is published. AI solutions, particularly LLMs, are now streamlining evidence‐generation and synthesis tasks. As these platforms mature, they promise to deliver a faster, more reliable flow of evidence for guideline developers and, ultimately, for patients.

LLMs are shrinking the interval between trial conception and first patient. A recent experiment has demonstrated that ChatGPT‐4 (OpenAI) produces protocol skeletons with objectives, eligibility criteria, and statistical plans, compressing weeks of authoring into hours.^96^ Similarly, ChatGPT‐4^97^ and Mistral 8x22B^98^ (Mistral AI) generated multilingual, plain‐language consent forms that meet US readability standards and regulatory key‐information requirements, reducing iterative changes with ethics committees. Clinical trial matching is another avenue of active research. Real‐time NLP engines mine EHRs to match complex protocol criteria against patient attributes.^99^, ^100^ Beyond static matching, machine‐learning–driven, predictive‐enrichment phenomapping identifies biologically homogeneous subgroups most likely to benefit from an investigational therapy, enabling adaptive eligibility refinements during the trial itself.^99^ To curb recruitment demands and ethical concerns about placebo exposure, sponsors increasingly build synthetic control cohorts from real‐world data or historical trials. A recent proof‐of‐concept study generated an entire cohort of synthetic patients with acute myeloid leukemia using generative AI and successfully reproduced the survival curves of a traditional control arm, highlighting double‐digit reductions in projected trial cost and duration without compromising fidelity.^101^

Given the rapid advances in available evidence, automated workflows to facilitate different steps of living‐evidence synthesis is an area of growing interest. LLMs can transform the practice of evidence synthesis by formulating a comprehensive literature search, screening relevant citations, extracting prespecified data elements, performing critical appraisal, and completing statistical analyses.^102^, ^103^, ^104^, ^105^, ^106^, ^107^ These models can support a framework of *living* practice guidelines that are updated on a near real‐time basis as new evidence becomes available.

## PATIENT‐REPORTED OUTCOMES

As cancer treatments prolong survival, focus has shifted from mere longevity to the cumulative burden of disease and treatment‐related comorbidities. Consequently, patient‐reported outcomes (PROs), such as health‐related quality of life, functional status, and self‐reported toxicities, have become core end points in both clinical trials and real‐world evidence studies. Yet traditional methods to capture PROs through clinic visits and through periodic surveys yield sporadic, heterogeneous data, limiting their usefulness for day‐to‐day management and policy decisions.

AI platforms now provide continuous, scalable capture and interpretation of PRO data.^108^ NLP pipelines can extract symptom information from free‐text clinic notes, patient diaries, and secure‐message threads^109^ while downstream machine‐learning models flag clinically meaningful deterioration and prompt timely intervention.^110^, ^111^ In multiple myeloma, for instance, a rule‐based NLP algorithm identified descriptors of fatigue, neuropathy, and bone pain in unstructured notes with near‐perfect agreement to manual abstraction (F1 = 0.95–0.99).^112^ Beyond cross‐sectional capture, AI supports longitudinal symptom monitoring.^110^ Examples include a hybrid model that combined weekly PROs (i.e., pruritus, pain, breast swelling) with serum cytokine levels and predicted grade ≥2 radiation dermatitis in patients with breast cancer before it became clinically evident, showcasing an area under the receiver operating characteristic curve of 0.78 (95% CI, 0.701–0.854).^113^ In oropharyngeal cancers, a systematic review demonstrated that combining longitudinal PROs (dysphagia, xerostomia, and pain) with radiomics features forecasted late toxicities of chemoradiotherapy, with the majority of algorithms reaching area under the receiver operating characteristic curve ≥0.80 for grade ≥2 dysphagia.^114^ At a population level, AI can harmonize disparate PRO instruments and impute missing data and surface trends that inform formulary decisions and supportive‐care resource allocation.

## FUTURE DIRECTIONS

The next decade will be defined by a shift from stand‐alone AI tools to an interoperable, continuously learning ecosystem. Multimodal, long‐context foundation models already accept text, images, and, increasingly, genomic data in a single prompt, allowing cross‐modal reasoning that links, for example, radiographic response with emerging resistance mutations.^115^, ^116^ In addition, digital‐twin platforms are beginning to simulate individual tumor trajectories and treatment courses, offering a sandbox for dose optimization or surgery–radiation sequencing before any real‐world intervention.^117^ Unlocking the data volume required for these advances will depend on privacy‐preserving federation and differential‐privacy overlays that let algorithms train across institutions without moving raw data, thereby increasing generalizability while satisfying data‐privacy guardrails.^118^

Clinically, AI will drive a new generation of adaptive, equity‐conscious research and care. Automated protocol drafting, real‐time EHR eligibility alerts, predictive enrichment phenomapping, and synthetic or real‐world external controls are converging to shorten trial timelines and lower costs, making truly adaptive oncology studies feasible even for rare disease cohorts.^119^, ^120^ To keep guidelines current, LLM‐assisted, *living* systematic‐review pipelines will feed a feedback loop in which new evidence, once published or posted, is screened, extracted, and meta‐analyzed with minimal human lag. At the point of care, richer PRO streams, ambient documentation, and multimodal decision support will enable human‐AI teams in which the system surfaces uncertainty, presents ranked rationales, and invites expert override rather than replacing clinician judgement.

Sustaining these gains will require robust governance, life‐cycle monitoring, and a commitment to equity and environmental stewardship.^121^ Regulatory frameworks, such as the US Food and Drug Administration's evolving CDS guidance and the European Union's AI Act are moving toward mandatory bias audits, subgroup performance reporting, and postdeployment drift detection, mirroring pharmacovigilance for drugs. Health‐system dashboards that track model performance, trigger automatic recalibration, and log clinician overrides will become standard infrastructure. Finally, the field must address the computational footprint of ever larger models through research into sparse architecture and on‐device inference. Investments in federated learning networks, bias‐remediation toolkits, digital‐twin validation consortia, and adaptive reimbursement schemes will ensure that the promise of AI translates into durable, equitable improvements for every patient living with or beyond cancer.

## CONCLUSION

**FIGURE 1:**
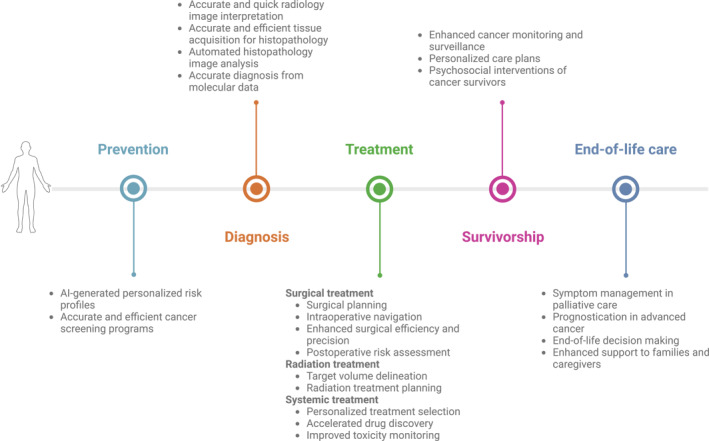
Application of artificial intelligence throughout the cancer continuum. Application of artificial intelligence in prevention, diagnosis, treatment, survivorship, and end‐of‐life care for patients with cancer. AI indicates artificial intelligence. Created in BioRender (Khan, M. [2025]; https://BioRender.com/wg1gqgf).

AI is rapidly transforming oncology, offering unprecedented opportunities to improve cancer care across the entire disease spectrum—from prevention and early detection to treatment, survivorship, and end‐of‐life care (Figure 1). AI‐powered tools enhance cancer risk assessment, optimize screening programs, refine diagnostic precision, and personalize treatment strategies, ultimately improving patient outcomes. Moreover, AI facilitates care delivery, streamlines evidence generation and syntheses, and optimizes the recording of PROs. In addition, AI‐driven advancements in fundamental biology, such as protein structure prediction and cancer genomics, are enhancing our understanding of the disease. At the same time, applications in robotic surgery, radiation therapy planning, drug discovery, and patient support are well poised to improve patient care and treatment outcomes.

However, realizing the full potential of AI in cancer care requires addressing health care workforce education^122^ and critical challenges, such as rigorous validation of AI algorithms, ensuring data privacy, mitigating algorithmic bias, and seamlessly integrating AI into clinical workflows.^123^, ^124^

Educating health care professionals on AI principles, applications, and limitations is crucial for successful implementation. By fostering confidence in AI tools and ensuring their role as decision‐support systems, we enable meaningful engagement between AI and clinicians. Human–AI collaboration is at the heart of AI's success in oncology—AI excels in data analysis and predictive modeling, but human expertise is essential for clinical decision making, patient communication, and ethical considerations. The synergy between AI insights and physician judgment enables more precise, personalized, and patient‐centered care, making this collaboration the key to transforming cancer treatment.

Multidisciplinary collaboration among clinicians, researchers, data scientists, and technology developers is vital to refining AI models, ensuring interpretability, and expanding AI applications in oncology. As AI continues to evolve and access to high‐quality data improves, its role in cancer care will become even more effective.

The future of oncology is increasingly intertwined with AI. By embracing this technology responsibly, educating health care professionals, and fostering human–AI collaboration, we can usher in a new era of personalized, precise, and effective cancer care—ultimately improving outcomes and quality of life for all patients.

## AUTHOR CONTRIBUTIONS

**Irbaz Bin Riaz**: Conceptualization; formal analysis; methodology; visualization; writing–original draft; writing–review and editing. **Muhammad Ali Khan**: Visualization; writing–original draft; writing–review and editing. **Travis Osterman**: Conceptualization; methodology; project administration; supervision; writing–original draft; writing–review and editing.

## CONFLICT OF INTEREST STATEMENT

Travis J. Osterman reports research support and/or grants/contracts from the Conquer Cancer Foundation, the Evelyn Selby Stead Fund for Innovation, GE Healthcare, IBM, Microsoft, the National Cancer Institute, and Outcomes4Me; consulting/personal and/or speaking fees from Amazon, the American Society of Clinical Oncology, European Society of Medical Oncology, AstraZeneca, Biodesix, Cota Healthcare, Dedham Group, MD Outlook, the National Comprehensive Cancer Network, and Outcomes Insights; and ownership of the business FacultyCoaching.com outside the submitted work. Irbaz Bin Riaz and Muhammad Ali Khan disclosed no conflicts of interest.

## Data Availability

Data are available in the article supporting information.
